# Executive Overconfidence, Digital Transformation and Environmental Innovation: The Role of Moderated Mediator

**DOI:** 10.3390/ijerph19105990

**Published:** 2022-05-14

**Authors:** Peiyan Zhou, Shuya Zhou, Ming Zhang, Shujuan Miao

**Affiliations:** School of Business and Management, Jilin University, Changchun 130012, China; zh-peiyan@163.com (P.Z.); vanilla5176@163.com (M.Z.); miaosj@jlu.edu.cn (S.M.)

**Keywords:** executive overconfidence, digital transformation, environmental technology innovation, environmental management innovation, industry competition, economic policy uncertainty, asset size, internal control

## Abstract

With the increasingly prominent energy and environmental problems, environmental innovation has become a critical path to achieving the goal of coordinating economic development and environmental protection fundamentally. This study aims to examine the impacts of executive overconfidence on environmental innovation and the mediating role of digital transformation. We conduct empirical tests based on the panel data of Chinese publicly listed enterprises during the period of 2007–2019. The results exhibit that (a) executive overconfidence can significantly promote environmental technology innovation but has no obvious effect on environmental management innovation; (b) executive overconfidence can significantly enhance digital transformation, and, accordingly, digital transformation can significantly promote environmental technology innovation and environmental management innovation; (c) industry competition and economic policy uncertainty can enhance the positive effect of executive overconfidence on digital transformation; and (d) a firms’ asset size can enhance the impact of digital transformation on environmental technology innovation; internal control positively moderates the impact of digital transformation on environmental technology innovation and negatively moderates the impact on environmental management innovation. This study not only breaks the stereotype about overconfidence and confirms its positive impact on digital transformation and environmental innovation but also provides insights for enterprises to improve environmental innovation through digital transformation.

## 1. Introduction

With the deterioration of the ecological environment, green development has gradually become the consensus of the international community. As the basic element of promoting green development, environmental innovation is the critical path to achieving the goal of coordinating economic development and environmental protection fundamentally. In recent years, governments have actively conducted a series of environmental supervision and incentive policies to alleviate environmental problems and promote environmental innovation, such as issuing environmental protection tax laws and granting environmental subsidies [1]. 

In addition, the infiltration of the green development concept has gradually changed consumer expectations, making the competitive advantage of green products increasingly prominent. To respond to external market pressures and meet internal values, increasing enterprises take the initiative to integrate environmental priority into their operational and innovative activities, which creates a favorable environment for environmental innovation [2,3]. Therefore, how to effectively enhance environmental innovation has become the focus of scholars and managers.

The extant literature has documented, in detail, the determinants of environmental innovation. Most of these studies focus on external factors that propel enterprise environmental innovation, such as environmental regulation [4], government subsidies [1], green credit [5], stock market liberalization [6], and economic policy uncertainty [7]. Only a few studies paid attention to organization-level factors, such as senior management experience [8], corporate governance [9], and digitalization [10]. However, compared with external pressure deterrence, the promotion of firms’ subjective initiative on environmental innovation should not be ignored [8]. 

Since environmental innovation is characterized by high investment, high risk, and dual externalities, enterprise executives lack the motivation to take the initiative to meet this huge challenge of high risk and low return [5,8]. Consequently, it is of great significance for scholars to extensively explore how to stimulate the subjective initiative of enterprise executives to commit to environmental innovation. At present, scholars have focused on the experience of executives based on imprinting theory. For instance, He et al. [8] and Zhao et al. [11] proved that the academic experience of senior management can positively influence environmental innovation. 

However, there is a paucity of research on the impact of executives’ psychological characteristics on environmental innovation. In fact, the psychological characteristics of managers, such as overconfidence, have long been introduced into the research field of management and finance. Scholars have proven that overconfidence can promote the innovation activities of enterprises [12]. It has been recognized that overconfident executives have optimistic and aggressive proactive personalities and are more willing to take risks and perform activities with high investment, high risk, and high uncertainty [13]. Given this, our study extends executive overconfidence to the research category of environmental innovation.

For innovation activities, executives’ subjective initiative is endowed with great significance; however, it is also worth exploring how executives can effectively promote environmental innovation. In recent years, quantum leaps in new digital technologies have changed the business model of enterprises and attracted extensive attention from managers and scholars [14]. According to Boeker et al. [15], advances in digital technologies facilitate the flow of knowledge, serving as a significant driver of innovation. 

Li and Shen [10] further confirmed that corporate digitalization can enhance internal supervision and promote green innovation levels. In addition, digital transformation enables enterprises to facilitate consumer participation and improve the matching degree between environmental innovation products and market demand [16]. Therefore, it can be inferred from these insights that digital transformation may be an effective approach to promote environmental innovation. 

Nevertheless, due to the inherent technological uncertainty and the universality of organizational changes, digital transformation also poses great challenges to executives. Although previous studies have focused on the significance of leader characteristics on digital transformation, these studies have not revealed the specific role of executives’ psychological characteristics on digital transformation. In an attempt to advance previous research, this paper delves into the relationship among executive overconfidence, digital transformation, and environmental innovation.

Along this line of thinking, we perform an empirical study to examine how overconfident executives enhance environmental innovation by promoting digital transformation. At first, we refer to Ren et al. [1] to classify environmental innovation as environmental technological innovation and environmental management innovation. Then, we conduct empirical tests using the panel data of Chinese publicly listed enterprises during the period 2007–2019. The main results indicate that executive overconfidence can significantly promote environmental technology innovation but has no obvious effect on environmental management innovation. 

The further mediating effect test suggests that executive overconfidence can significantly improve the level of digital transformation, and digital transformation can also significantly promote environmental technology innovation and environmental management innovation. The contradiction between the total effect and the mediating effect of executive overconfidence on environmental management innovation may be due to the existence of an undetected masking effect. 

In general, the above results confirm that digital transformation is an effective path for executives to promoting environmental innovation, including environmental technology innovation and environmental management innovation. Our primary results remain robust after performing a series of endogeneity and sensitivity tests. Next, we further explore the moderating effects of some contingent factors. Given the external pressure and information asymmetry brought by industry competition and economic policy uncertainty [17,18], our empirical results indicate that industry competition and economic policy uncertainty can enhance the positive effect of executive overconfidence on digital transformation. 

In addition, due to the dependence and competitiveness of digital orientation and environment orientation on resources [2,19], we introduce the firm asset size as a moderating variable. We also examine the moderating role of internal control because of its profound impact on firm economic activities [10,20]. The results suggest that the firm asset size and internal control quality can enhance the positive effect of digital transformation on environmental technology innovation. However, for the impact of digital transformation on environmental management innovation, the moderating effect of asset size is not significant, and internal control is negative.

Our study contributes to the literature in the following ways. First, we reveal the influence channels of executive psychological characteristics on environmental innovation. Most previous studies proposed that executive overconfidence has a negative effect on enterprise performance; however, our study breaks this view and affirms the positive effect of such psychological traits on digital transformation and environmental innovation. 

Second, we contribute to the research on environmental innovation by elaborating on the impact of executive overconfidence and digital transformation on environmental innovation. Third, our study provides insights for enterprises to improve digital transformation. At the same time, we also verify the positive effect of digital transformation on environmental innovation, which enriches the research on the impact of digital transformation.

The remainder of this study is structured as follows: Section 2 reviews the existing literature and derives hypotheses. Section 3 elaborates the research design. Section 4 provides the main empirical results and further analysis. Section 5 discusses the main results and implications. Section 6 provides our conclusions and limitations.

## 2. Literature Review and Hypothesis Development

In recent years, due to the external environmental regulatory pressure and the emergence of green consumption conception, environmental innovation has become a pivotal component of enterprise innovation activities, which has attracted extensive attention from scholars [1,4,21]. It has been widely recognized that environmental innovation refers to any product, process, management, or organizational innovation aimed at mitigating environmental burden [1,21,22]. Different from general innovation, environmental innovation has double externalities, including not only common knowledge spillover but also environmental externalities [22,23]. 

In order to release this double dividend, on the one hand, enterprises need to actively adopt environmental innovation technology to replace the traditional technology with high pollution and high energy consumption, which is conducive to promoting sustainable development [22]. On the other hand, enterprises also need to develop and implement a series of management initiatives to promote environmental performance [24]. 

Given that, we refer to the practice of Ren et al. [1] to classify these two types of innovation as environmental technology innovation and environmental management innovation. Environmental technology innovation is the technological innovation related to the environment, usually measured by the number of green innovation patent applications [1]. Environmental management innovation refers to the innovation of a series of management activities aimed at improving environmental performance, such as training employees on specific environmental operation steps, usually measured by the use of environmental management systems (e.g., ISO 14001 environmental management certification) [1,25].

As for digital transformation, the existing literature has well-documented its necessity for enterprise innovation in the digital era [26,27]. It is not only about adopting a portfolio of digital technologies to optimize existing business processes but also about capturing new digital business models to create more value for enterprises [26,28,29]. According to Verhoef et al. [29], digital transformation is a continuous innovation from digitization, and digitalization to digital transformation, with each phase requiring the alignment of specific digital resources, organizational structure, digital growth strategies, as well as metrics and goals. Such a process requires the skillsets and mindsets of managers and employees to be aligned to gain new skills and knowledge [30]. Consequently, as the main decision-makers of enterprise strategic planning and resource allocation, executives play a vital role in promoting digital transformation and the environmental innovations of enterprises [8,11]. However, there is still a lack of research on how the characteristics of executives influence environmental innovation through promoting digital transformation.

Based on these backgrounds, the following part mainly analyses (i) the relationships among executive overconfidence, digital transformation, and environmental innovation; and (ii) the moderating factors affecting the relationships among executive overconfidence, digital transformation, and environmental innovation.

### 2.1. Executive Overconfidence and Environmental Innovation

Due to the urgency of environmental protection, a substantial body of research has explored the influencing factors of environmental innovation from various perspectives. For example, at the macro-level, some scholars confirmed that environmental regulations, subsidies, and green credit can affect environmental innovation [1,4,7]. At the micro-level, some scholars proved that corporate governance and supply chain management also have impacts on environmental innovation [9,31]. 

Since the characteristics of high investment, high risk, and dual externalities of environmental innovation pose huge challenges to enterprise executives, a growing number of scholars have begun to link executive characteristics with environmental innovation. For instance, He et al. [8] affirmed that senior executives’ academic experience can promote environmental technology innovation. Galbreath [32] discovered that female executives are more sensitive to environmental issues and thus more likely to promote environmental innovation.

According to upper echelon theory, organizational complex decisions, to a large extent, reflect the values and cognitive bases of the firm’s powerful actors [33]. CEOs, who are the primary decision-makers in businesses, are supposed to inevitably inject their traits into the decision-making process due to their bounded rationality. The extant literature documents that executives’ backgrounds, personal attributes, and leadership styles have profound impacts on the organizational strategy and effectiveness [12,33]. For instance, the prominent characteristics of CEOs, such as age, gender, educational background, professional experience, and financial position, are appropriate proxies of psychological and behavioral traits, which further affect the output results [34,35]. Among these characteristics, executive overconfidence, as a typical psychological personality, has been proven to be crucial for innovation activities [12,36]. In general, overconfident managers tend to rise to challenges and are more willing to take on risky projects, such as research and development. 

Since environmental innovation refers to the combination of environmental orientation and enterprise innovation, it has the same complexity and uncertainty as does general innovation [1]. Consequently, in the face of stakeholder interest demands and expectations of ecological protection, overconfident executives tend to address these challenges with optimism and initiative. They are prone to engage in environmental innovation to thereby convey positive signals to the market and gain recognition from stakeholders [12]. In addition, executives’ optimism about their capabilities can hedge their concerns about projects not going well or financing constraints, greatly reducing the resistance to environmental innovation [36]. In light of this, we speculate that executive overconfidence can positively affect environmental innovation. Thus, we propose the following hypotheses:

**Hypothesis** **1a** **(H1a).** *Executive overconfidence positively relates to environmental technology innovation.*

**Hypothesis** **1b** **(H1b).** *Executive overconfidence positively relates to environmental management innovation*.

### 2.2. Executive Overconfidence and Digital Transformation

The infusion of digital technologies and the volatile competitive environment force enterprises to seek sustainable competitive advantages through digital transformation [37]. However, due to the inherent technological uncertainty and the universality of organizational changes, digital transformation not only provides ample market opportunities for enterprises but also brings huge management challenges [38]. Therefore, the skill and personal characteristics of executives are crucial for enterprises to implement digital transformation [39,40]. 

Notably, digital transformation is an organization-wide strategic decision with substantial risks that requires enormous resources and time to implement [41]. Since overconfident executives have optimistic and aggressive proactive personalities, they are more willing to take risks and perform activities with high investment, high risk, and high uncertainty [13]. Compared with non-overconfident executives, overconfident executives tend to underestimate the potential risks of digital transformation and optimistically anticipate the long-term benefits, which provides them with the powerful impetus to overcome the resistance to digital transformation [42]. 

Furthermore, the great challenges of system construction and organizational reconfiguration triggered by digital transformation led to higher requirements for executives’ digital skillsets and knowledge absorption capacity [43]. Correspondingly, overconfident executives are more likely to have the courage and determination to tackle these challenges and be open to learning new knowledge and skills needed for transformation [36]. Based on these arguments, we develop the following hypothesis:

**Hypothesis** **2** **(H2).** *Executive Overconfidence positively relates to the digital transformation of enterprises.*

### 2.3. Executive Overconfidence, Digital Transformation, and Environmental Innovation

According to the foregoing discussion, overconfident executives are inclined to engage in environmental innovation activities due to the pressure from stakeholders on the one hand and their risk preference on the other. However, how executives effectively promote environmental innovation requires further exploration from scholars. In recent years, with the advancement of digital technologies, an increasing number of studies have begun to focus on how to adopt digital technology to facilitate environmental innovation. For example, Gupta et al. [44] proved that Cloud-based Enterprise Resource Planning (Cloud ERP) can maximize resource utilization and positively impact environmental performance. Joerß et al. [45] confirmed that augmented-reality-based recommendation agents (AR-RAs) can stimulate consumers’ environmental awareness in the process of shopping. Furthermore, Li and Shen [10] verified that corporate digitalization can enhance internal supervision and promote green innovation levels. Building on the previous literature, we conjecture that digital transformation is an effective way for executives to promote environmental innovation.

First, digital transformation can help enterprises facilitate consumer participation and improve the matching degree between enterprises’ environmental innovation outcomes and consumer demand [16]. Due to the positive externalities of environmental innovation, enterprises mainly innovate under the pressure of external supervision [1] but lack internal motivation for innovation. Thus, it is difficult to transform the innovation achievements into market value to compensate for compliance costs. 

However, digital transformation makes it possible to obtain valuable insights into customers’ unique and specific needs or allow customers to choose options and configure the product according to their specifications through a digital interface [46]. Compared with standardized products, personalized products can better meet the needs of users and create higher value for both users and enterprises, thus, improving the internal motivation for environmental innovation [47].

Second, digital transformation promotes resource integration and knowledge sharing, which provides a flexible and open innovation environment [15,48,49]. Since the innovation process of environmentally friendly products is a relatively complex activity, it entails resource-intensive inputs and complex technology adoption [9]. Digital transformation enables enterprises to deploy networked resource allocation and digital technology empowerment [10]. For example, the application of cloud ERP can connect organizational functions and resources in real-time on the cloud platform, thus, reducing information loss and maximizing the use of innovative resources [44]. Intelligent and automated production lines enabled by digital technology can realize full monitoring of the production process, making it possible to quickly identify quality defects and high energy consumption links [10]. Real-time data generated by intelligent manufacturing processes further supports the decision-making of green innovation projects. In addition, digital transformation enables knowledge flow to break through the limitations of time and region, thus, accelerating knowledge sharing within and across technology fields [15]. By enhancing the acquisition and integration of internal and external knowledge, digital transformation provides enterprises with the source power needed for environmental innovation.

Third, digital transformation can enhance corporate governance and improve the efficiency of innovation management by reducing information asymmetry and restructuring organizational structure [10]. According to Amore and Bennedsen [9], managers tend to avoid cognitively challenging or systemically destructive activities in order to maximize their personal interests, which inhibits enterprises’ environmental innovation activities. 

However, in the digital context, the adoption of digital technologies, such as mobile devices, 5G, and electronic data management systems, promotes the flow of information and improves the transparency of enterprise management [50]. To a certain extent, the improved corporate governance enhances the managerial effort to invest in environmental innovation [9]. On the other hand, digital transformation ultimately leads to the development of new business models, involving the redesign of internal processes [29]. If the process of restructuring is integrated with environmental orientation, it will substantially promote environmental management innovation.

Taken together, we contend that digital transformation can affect environmental innovation. According to the previous discussion of H1 and H2, we can propose that overconfident executives can indirectly promote environmental innovation by implementing digital transformation. Thus, we formulate the following hypotheses:

**Hypothesis** **3a** **(H3a).** *Digital Transformation is positively correlated with firms’ environmental technology innovation and mediates the impact of executive overconfidence on environmental technology innovation*.

**Hypothesis** **3b** **(H3b).** *Digital Transformation is positively correlated with firms’ environmental management innovation and mediates the impact of executive overconfidence on environmental management innovation*.

### 2.4. Moderating Factors between Executive Overconfidence and Digital Transformation

Previous literature has demonstrated that fierce competition makes enterprises more entrepreneurial to embrace digital transformation [17]. The technological revolution has not only changed consumer expectations and behavior but also reshuffled the landscape of the market [29]. In turn, increasing competitive pressure makes it more urgent for enterprises to seek new competitive advantages through digital transformation [49]. At the same time, due to the inherent characteristics of high investment, long cycles, and wide ranges, digital transformation also brings great challenges to enterprises [38]. 

In the face of keen market competition and challenges, overconfident executives are more motivated to demonstrate their competence through digital transformation. By contrast, in a less competitive environment, executives have less motivation to change the status quo because the survival of enterprises is less threatened from the outside. In this vein, industrial competition is the catalyst for the relationship between executive overconfidence and enterprise digital transformation. The more intense the competition, the more inclined executive overconfidence will be to invest in digital construction to gain core competitiveness. Hence, we predict the following hypothesis:

**Hypothesis** **4** **(H4).** *The effect of executive overconfidence on digital transformation is strengthened by industrial competition.*

Recent studies often extend the effects of economic policy uncertainty to firm-level economic activity [7,51]. To some extent, the uncertainty of economic policy constrains the market expansion and M&A activity of enterprises [7,51]. In particular, the uncertainty of economic policy intensifies the asymmetry of market information and increases the risk of enterprise operation [18], whereas digital transformation enhances the information processing capability and market competitiveness of enterprises by developing new business models [29]. In search of new market growth, overconfident executives may be more aggressive in initiating an organization-wide digital revolution to create more value for firms. In view of this, we assert the following hypothesis:

**Hypothesis** **5** **(H5).** *The effect of executive overconfidence on digital transformation is strengthened by economic policy uncertainty*.

### 2.5. Moderating Factor between Digital Transformation and Environmental Innovation

Based on resource-based theory, innovation performance is largely constrained by resource endowment and strategic orientation [52]. Compared with general innovation, environmental innovation is more prone to rely on the commitment of scarce and irreplaceable resources due to its dual externalities [2]. Although the application of digital technology theoretically improves the efficiency of environmental innovation, resources are also indispensable to the implementation of digital transformation. 

Due to the limitation of resources, enterprise managers may not be able to meet the resource commitment of digital transformation and environment orientation simultaneously [2,19]. Especially for small and medium-sized enterprises, pursuing both digital orientation and environmental orientation may cause employees to face overload tasks, knowledge, and stakeholder interactions, thus, counteracting the positive impact of digital transformation on environmental innovation [2]. As such, we foretell the following hypotheses:

**Hypothesis** **6a** **(H6a).** *The effect of digital transformation on environmental technology innovation is more prominent in firms with higher assets*.

**Hypothesis** **6b** **(H6b).** *The effect of digital transformation on environmental management innovation is more prominent in firms with higher assets*.

As the control process of enterprise operation and management, internal control has a profound influence on the economic activities of enterprises. Effective internal control can help enterprises improve information transparency and strengthen supervision and incentive for managers [53]. Since environmental technology innovation is an economic activity with high investment and high risk, managers may restrain innovation input for the pursuit of personal interests, thereby, resulting in agency costs. 

However, high-quality internal control can restrain managers’ opportunism to some extent and make them more likely to make decisions conducive to the promotion of the long-term interests of enterprises [10,20]. From this perspective, effective internal control can reduce the resistance of enterprises to promote environmental technology innovation through digital transformation. We thus posit the following hypothesis:

**Hypothesis** **7a** **(H7a).** *The effect of digital transformation on environmental technology innovation is more prominent in firms with higher internal control quality*.

According to the foregoing discussion, digital transformation can enhance corporate governance and optimize management processes, thereby, promoting innovation in environmental management. Hence, in the absence of internal control, digital transformation can make up for the defects of internal control and promote enterprise environmental innovation more effectively [10]. However, in a high-quality internal control environment, the agency problem affecting environmental management innovation can be effectively suppressed, and the management efficiency can be greatly improved. Therefore, the positive effect of digital transformation on environmental management innovation may not be obvious under high-quality internal control. In line with this reasoning, we propose the following hypothesis:

**Hypothesis** **7b** **(H7b).** *The effect of digital transformation on environmental management innovation is more prominent in firms with lower internal control quality*.

**Figure 1:**
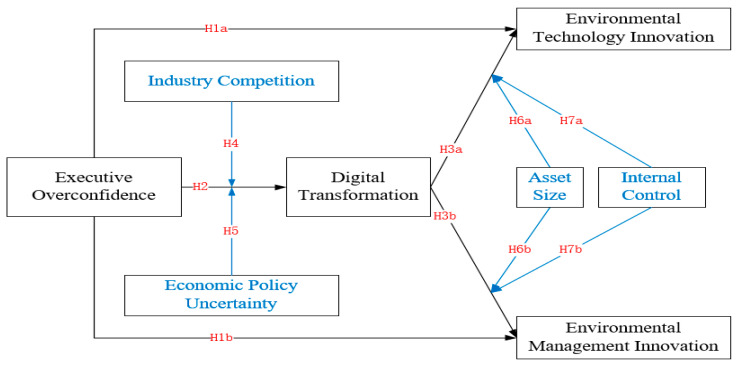
Research design.

The technical roadmap of this paper is as follows (Figure 1):

## 3. Research Design

### 3.1. Sample and Data Collection

We collected panel data from listed companies in the Shanghai and Shenzhen A-share markets in China as research samples. Empirical evidence from China provides an ideal natural setting for testing our framework. First, as a developing country, China has been facing serious environmental pollution and resource depletion in the past, and thus it is undergoing an economic transition from relying on heavily polluting industries to a green economy. An increasing number of firms start to go green, which provides a quasi-natural environment for green innovation research. Second, China’s booming digital economy and complete digital infrastructure provide a broad platform for the digital transformation of various industries. The intersection and integration of digital technology and energy technology also mean that the digital economy has broad application prospects in the field of green development. In summary, the Chinese business environment allows us to observe many variations in the relationships among executive overconfidence, digital transformation, and environmental innovation.

The sample period that we selected spans 13 years, from 2007 to 2019. We chose 2007 as the start year because China implemented new accounting standards on 1 January 2007, resulting in the incomparability of annual data between the years before and after the change. Additionally, we chose 2019 as the end year because COVID-19, as a special event, may influence the process of firms’ digital transformation after 2020 [54].

There are four data sources: First, we manually collected executives’ personal information and the digital transformation data from listed companies’ annual reports. Second, we obtained the patent classification number information of all listed companies from the Chinese Research Data Services Platform (CNRDS) and matched it with the Green List of International Patent Classification (Green List) issued by The World Intellectual Property Organization (WIPO) in 2010. We then calculated the number of green patent applications of enterprises according to the matching results. Third, data on ISO 14001 environmental management certification were collected from the Certification and Accreditation Administration of the People’s Republic of China. Fourth, other financial and accounting data were adopted from the China Stock Market and Accounting Research (CSMAR) database, a reliable source for collecting China-listed firm data. 

To improve the data quality, we further performed strict screening on the sample data. First, we excluded firms with Special Treatment or Particular Transfer (ST/PT) for the reason that their financial data may deviate from normal values. Second, we excluded financial firms (e.g., banks, insurance companies, and investment trusts) as they are subject to different accounting and reporting rules and tend to have a capital structure different from other companies [8,55]. Third, we further excluded observations with missing variable values. After applying the minimum data filtering, we were left with a sample of 22,989 firm-year observations for the remaining 3213 companies (see Table A1 in Appendix A). In order to avoid the influence of outliers, we performed 1%winsorize bilateral tailing for continuous variables.

### 3.2. Variable Measures

#### 3.2.1. Independent Variable: Executive Overconfidence (*OC*)

According to Chen et al. [56], overconfidence refers to the phenomenon that executives tend to overestimate their ability or skills on the one hand and the accuracy of their judgment on the other hand. Previous studies have demonstrated that CEOs of younger ages, male gender, and higher education level are prone to be overconfident and willing to take risks of transformation and innovation [57,58,59]. In addition, CEOs who are also chairmen, or have research and development backgrounds, or professional technical backgrounds tend to overestimate their abilities due to their own professional or educational experiences, further contributing to their overconfidence [60,61]. 

In view of these, we followed Li and Zhang [12] and used six characteristics of CEOs, including age, gender, formal education level, position, expertise background, and experience background, to construct the score of executive overconfidence. We assigned binary values to each characteristic indicator. If the CEO is younger than the average, male, has a graduate degree or above, is also the chairman, and has R&D experience and technical professional background, each corresponding indicator is assigned a value of 1; otherwise, 0. Finally, if the sum of the six indicators exceeds 3, it means that the CEO is overconfident, and the dummy variable of executive overconfidence (*OC*) is 1, otherwise it is 0.

#### 3.2.2. Dependent Variables: Environmental Innovation

Based on Ren et al. [1], we adopted environmental technology innovation (*ETI*) and environmental management innovation (*EMI*) as the proxy variables of environmental innovation. First, we used environmental technology patent applications to represent *ETI*. Since the number of patent applications reflects the output of innovation activities and is publicly available for long time series, it has been adopted by many scholars to measure the innovation level of enterprises [62]. Therefore, we selected from the patent database the patents that achieve energy conservation and emission reduction by improving product design and production process, or switching to green energy, and then counted the annual number of green patent applications of each enterprise. To make the data more consistent with the assumption of normal distribution, we used the natural logarithm of one plus the number of green patent applications as the proxy variable of *ETI*. 

Second, we measured the level of *EMI* according to whether enterprises have the ISO 14001 environmental management certification. As ISO 14001 certification requires enterprises to develop internal environmental standards, goals, and performance indicators, it reflects the environmental management level of enterprises. Hence, *EMI* is assigned a value of 1 if the enterprise has ISO14001 certification in a given year and 0 otherwise.

#### 3.2.3. Mediating Variable: Digital Transformation (*DIG*)

Referring to the practice of Zhou et al. [54], we used the combination of digital technologies to measure the degree of digital transformation. Based on Zhou et al. [54], we classified the use of digital technologies into the following items: Artificial Intelligence, Blockchain, Data Management, Multichannel, and Digital infrastructures. 

Then, we determined the basic ways in which different enterprises expressed the items of digital transformation in the annual report through manual sorting and automatic word segmentation of Python algorithm. We used manual filtering and a computer associative structure algorithm to expand the keywords. Finally, we used the method of text mining to extract 30 words before and after the selected keywords from the annual report of the listed company in China, and conducted word frequency analysis and manual inspection to analyze whether the enterprise has adopted the corresponding digital technology. The items and selected keywords are presented in Table 1.

Based on the results of the above analysis, we treated each term as a binary variable. If the word frequency analysis results in the annual report showed that the company adopted the corresponding digital technology, the value is 1, otherwise it is 0. Then, we added up all the items. As a result, a value of 5 shows that the enterprise has adopted all digital technologies, indicating the highest level of digital transformation, while a value of 0 indicates that the firm has adopted none of these digital technologies, denoting the lowest level of digital transformation.

#### 3.2.4. Control Variables

Referring to previous studies [1,10,11,63], we controlled for a range of firm-level and country-level factors that might affect firms’ digital transformation and environmental innovation.

Firm-level factors include corporate economic characteristics and corporate governance variables, which can reduce the impact of possible omitted variables on the empirical results. Specifically, these variables involve the ratio of total liabilities to total assets at the end of the fiscal year (*LEV*), the ratio of net income to total assets at the end of the fiscal year (*ROA*), the ratio of change in current year’s sales revenue relative to last year’s sales revenue (*Growth*), the ratio of cash and equivalents to total assets at the end of the fiscal year (*CASH*), the ratio of market value to book value at the end of the fiscal year (*Tobin’s Q*), the ownership property (*SOE*), the natural log of firm’s age (*AGE*), duality (*DUAL*), the percentage of independent directors on the board (*IND*), and the natural log of the board size (*BOARD*).

At the country level, we controlled for the percentage change from the preceding period in per capita real GDP (*GDP*). The pursuit of GDP may affect the intensity of government environmental supervision on enterprises and thus affect environmental innovation. We also controlled the natural log of the level of marketization in the listed company’s province (*Market*). Areas with a higher level of marketization usually have higher requirements for environmental protection of enterprises, thus, prompting enterprises to participate in environmental innovation [64]. 

In addition, we included year and industry fixed effects to control for potential heterogeneity at these levels. The industrial classification is based on the criteria provided by the China Securities Regulatory Commission (CSRC). Detailed variable definitions are listed in Table 2.

### 3.3. Regression Models

To investigate the relationships among executive overconfidence (*OC*), digital transformation (*DIG*) and environmental innovation (*ETI*/*EMI*), we adopted the OLS method to establish the multiple linear regression models (1) to (8).

Model (1) was constructed based on H1 to detect the impact of executive overconfidence on environmental innovation. If the main coefficient $\beta_{1}$ of *OC* is significantly positive, it would confirm that executive confidence can improve environmental innovation.

(1) $$\begin{array}{cc} ETI_{i.t}\left(EMI_{i.t}\right)= & \beta_{0}+\beta_{1}OC_{i,t}+\beta_{2}LEV_{i,t}+\beta_{3}ROA_{i,t}+\beta_{4}Growth_{i,t}+\beta_{5}Cash_{i,t}+\beta_{6}\;Tobin’s\;Q_{i,t}+\beta_{7}\;SOE_{i,t}\\ & +\beta_{8}\;AGE_{i,t}+\beta_{9}\;DUAL_{i,t}+\beta_{10}\;IND_{i,t}+\beta_{11}\;BOARD_{i,t}+\beta_{12}\;GDP_{i,t}+\beta_{13}\;Market_{i,t}+Industry\;FE\\ & +Year\;FE+\varepsilon_{i,t} \end{array}$$

Model (2) was constructed based on H2 to detect the impact of executive overconfidence on digital transformation. If the main coefficient $\beta_{1}$ of *OC* is significantly positive, it would confirm that executive confidence can improve digital transformation.

(2) $$\begin{array}{cc} DIG_{i.t}= & \beta_{0}+\beta_{1}OC_{i,t}+\beta_{2}LEV_{i,t}+\beta_{3}ROA_{i,t}+\beta_{4}Growth_{i,t}+\beta_{5}Cash_{i,t}+\beta_{6}\;Tobin’s\;Q_{i,t}+\beta_{7}\;SOE_{i,t}\\ & +\beta_{8}\;AGE_{i,t}+\beta_{9}\;DUAL_{i,t}+\beta_{10}\;IND_{i,t}+\beta_{11}\;BOARD_{i,t}+\beta_{12}\;GDP_{i,t}+\beta_{13}\;Market_{i,t}\\ & +Industry\;FE+Year\;FE+\varepsilon_{i,t} \end{array}$$

Model (3) and (4) were constructed based on H3. Among them, model (3) can verify the impact of digital transformation on environmental innovation. If the main coefficient $\beta_{1}$ of *DIG* is significantly positive, it would indicate that digital transformation can promote environmental innovation. 

We constructed model (4) by referring to the practice of Baron and Kenny [65]. If the main coefficient $\beta_{2}$ of *DIG* is significantly positive, it would indicate that digital transformation is the intermediary of the impact of executive overconfidence on environmental innovation.

(3) $$\begin{array}{cc} ETI_{i.t}\left(EMI_{i.t}\right)= & \beta_{0}+\beta_{1}DIG_{i,t}+\beta_{2}LEV_{i,t}+\beta_{3}ROA_{i,t}+\beta_{4}Growth_{i,t}+\beta_{5}Cash_{i,t}+\beta_{6}\;Tobin’s\;Q_{i,t}\\ & +\beta_{7}\;SOE_{i,t}+\beta_{8}\;AGE_{i,t}+\beta_{9}\;DUAL_{i,t}+\beta_{10}\;IND_{i,t}+\beta_{11}\;BOARD_{i,t}+\beta_{12}\;GDP_{i,t}\\ & +\beta_{13}\;Market_{i,t}+Industry\;FE+Year\;FE+\varepsilon_{i,t} \end{array}$$

(4) $$\begin{array}{cc} ETI_{i.t}\left(EMI_{i.t}\right)= & \beta_{0}+\beta_{1}OC_{i,t}+\beta_{2}DIG_{i,t}+\beta_{3}LEV_{i,t}+\beta_{4}ROA_{i,t}+\beta_{5}Growth_{i,t}+\beta_{6}\;Cash_{i,t}\\ & +\beta_{7}\;Tobin’s\;Q_{i,t}+\beta_{8}\;SOE_{i,t}+\beta_{9}\;AGE_{i,t}+\beta_{10}\;DUAL_{i,t}+\beta_{11}\;IND_{i,t}+\beta_{12}\;BOARD_{i,t}\\ & +\beta_{13}\;GDP_{i,t}+\beta_{14}\;Market_{i,t}+Industry\;FE+Year\;FE+\varepsilon_{i,t} \end{array}$$

To further verify the moderating factors between executive overconfidence and digital transformation, we constructed model (5) and (6). First, we followed Zou et al. [66] and used the Herfindahl–Hirschman Index (*HHI*) to measure industrial competition. A higher *HHI* means less competition in the market. Then, we introduced the intersection term $OC_{i,t}\times HHI_{i,t}$ of overconfidence and industrial competition in model (5). If the coefficient $\beta_{2}$ in model (5) is significantly negative, The H4 about the moderating effect of industrial competition would be supported.

To test the moderating effect of economic policy uncertainty (*EPU*) proposed by H5, we referred to Baker et al. [67] and used the mean value of the monthly economic policy uncertainty index to measure the *EPU*. Then, we introduced the intersection term $OC_{i,t}\times EPU_{i,t}$ of overconfidence and economic policy uncertainty in model (6). The coefficient $\beta_{2}$ in model (6) was expected to be significantly positive if H5 was confirmed.

(5) $$\begin{array}{cc} DIG_{i.t}= & \beta_{0}+\beta_{1}OC_{i,t}+\beta_{2}OC_{i,t}\times HHI_{i,t}+\beta_{3}HHI_{i,t}+\beta_{4}LEV_{i,t}+\beta_{5}ROA_{i,t}+\beta_{6}Growth_{i,t}+\beta_{7}\;Cash_{i,t}\\ & +\beta_{8}\;Tobin’s\;Q_{i,t}+\beta_{9}\;SOE_{i,t}+\beta_{10}\;AGE_{i,t}+\beta_{11}\;DUAL_{i,t}+\beta_{12}\;IND_{i,t}+\beta_{13}\;BOARD_{i,t}\\ & +\beta_{14}\;GDP_{i,t}+\beta_{15}\;Market_{i,t}+Industry\;FE+Year\;FE+\varepsilon_{i,t} \end{array}$$

(6) $$\begin{array}{cc} DIG_{i.t}= & \beta_{0}+\beta_{1}OC_{i,t}+\beta_{2}OC_{i,t}\times EPU_{i,t}+\beta_{3}EPU_{i,t}+\beta_{4}LEV_{i,t}+\beta_{5}ROA_{i,t}+\beta_{6}Growth_{i,t}+\beta_{7}\;Cash_{i,t}\\ & +\beta_{8}\;Tobin’s\;Q_{i,t}+\beta_{9}\;SOE_{i,t}+\beta_{10}\;AGE_{i,t}+\beta_{11}\;DUAL_{i,t}+\beta_{12}\;IND_{i,t}+\beta_{13}\;BOARD_{i,t}\\ & +\beta_{14}\;GDP_{i,t}+\beta_{15}\;Market_{i,t}+Industry\;FE+Year\;FE+\varepsilon_{i,t} \end{array}$$

To further explore the moderating factors between digital transformation and environmental innovation, we constructed models (7) and (8). First, we used *SIZE* to represent the natural logarithm of firms’ assets. Then, we introduced the intersection term $DIG_{i,t}\times SIZE_{i,t}$ in model (7).

Second, we adopted Shenzhen DIB’s internal control indexes to measure internal control quality (*ICQ*). Then, we introduced the intersection term $DIG_{i,t}\times ICQ_{i,t}$ in model (8).

(7) $$\begin{array}{cc} ETI_{i.t}\left(EMI_{i.t}\right)= & \beta_{0}+\beta_{1}DIG_{i,t}+\beta_{2}DIG_{i,t}\times SIZE_{i,t}+\beta_{3}SIZE_{i,t}+\beta_{4}LEV_{i,t}+\beta_{5}ROA_{i,t}+\beta_{6}Growth_{i,t}\\ & +\beta_{7}\;Cash_{i,t}+\beta_{8}\;Tobin’s\;Q_{i,t}+\beta_{9}\;SOE_{i,t}+\beta_{10}\;AGE_{i,t}+\beta_{11}\;DUAL_{i,t}+\beta_{12}\;IND_{i,t}\\ & +\beta_{13}\;BOARD_{i,t}+\beta_{14}\;GDP_{i,t}+\beta_{15}\;Market_{i,t}+Industry\;FE+Year\;FE+\varepsilon_{i,t} \end{array}$$

(8) $$\begin{array}{cc} ETI_{i.t}\left(EMI_{i.t}\right)= & \beta_{0}+\beta_{1}DIG_{i,t}+\beta_{2}DIG_{i,t}\times ICQ_{i,t}+\beta_{3}ICQ_{i,t}+\beta_{4}LEV_{i,t}+\beta_{5}ROA_{i,t}+\beta_{6}Growth_{i,t}\\ & +\beta_{7}\;Cash_{i,t}+\beta_{8}\;Tobin’s\;Q_{i,t}+\beta_{9}\;SOE_{i,t}+\beta_{10}\;AGE_{i,t}+\beta_{11}\;DUAL_{i,t}+\beta_{12}\;IND_{i,t}\\ & +\beta_{13}\;BOARD_{i,t}+\beta_{14}\;GDP_{i,t}+\beta_{15}\;Market_{i,t}+Industry\;FE+Year\;FE+\varepsilon_{i,t} \end{array}$$

## 4. Empirical Results

### 4.1. Descriptive Statistics

Table 3 (Panel A) presents descriptive statistical results for all relevant variables. The mean value of *OC* is 0.267, indicating that only 26.7% of executives in the sample are overconfident. The mean value of *DIG* is 0.785 with a standard deviation of 1.085, indicating that the degree of firms’ digital transformation is still at a low level and shows considerable variation. 

As for environmental innovation, the standard deviation of *ETI* is 1.165, whilst the inter-quartile spread ranges from 0 to 4.522, indicating significant variation in the level of environmental technology innovation among our sample. The mean value of *EMI* is 0.198, indicating that only 20% of enterprises in the sample have obtained ISO14001 certification through environmental management innovation.

Table 3 (Panel B and C) provides the Pearson’s correlations matrix for all relevant variables. *OC* is positively correlated with *DIG*, *ETI*, and *EMI*, preliminarily suggesting that executive overconfidence can significantly promote digital transformation and environmental innovation. In addition, *DIG* is positively correlated with *ETI* and *EMI*, indicating that digital transformation can also enhance environmental innovation. In general, the majority of the correlations between variables are significant but small in magnitude. Since correlations between all variables are below 0.5, it can be judged that there is no serious multicollinearity among regression model variables.

### 4.2. Regression Results

Table 4 displays the main empirical results, in which control variables, annual effects, and industry effects are all included. Specifically, Columns (1) and (2) of Table 4 present the regression output for the effect of executive overconfidence on environmental innovation. As shown in Column (1) of Table 4, the coefficient of *OC* is positive and statistically significant at the 1% level (*β* = 0.211, *t* = 10.97), confirming that overconfident executives are more capable of promoting enterprise environmental technology innovation. However, the coefficient of *OC* in Column (2) of Table 4 is not statistically significant, indicating that executive overconfidence cannot effectively promote environmental management innovation. These results are consistent with H1a and inconsistent with H1b.

Column (3) of Table 4 shows the regression result of the impact of executive overconfidence on digital transformation. The key coefficient of *OC* is 0.258 (*t* = 15.44), which is significant and positive at the 1% level, indicating that executive overconfidence can effectively enhance the level of digital transformation. This result is economically significant: firms with overconfident executives saw a significant improvement of about 25.8% in digital transformation compared to those without overconfident executives. Thus, our prediction in H2 is supported.

Columns (4) and (5) of Table 4 display the regression results of the effect of digital transformation on environmental technology innovation (*ETI*) and the mediating role of digital transformation. As shown in Column (4), the coefficient of *DIG* is 0.196 (*t* = 21.84), which is statistically positive and significant at the 1% level. This result is consistent with the research of Li and Shen [10], which confirms that the implementation of digital transformation can promote environmental technology innovation. When the variable *DIG* is further added, the regression result shown in Column (5) of Table 4 exhibits that the coefficients of *OC* and *DIG* are both significantly positive at the 1% level (*OC*: *β* = 0.162, *t* = 8.56; *DIG*: *β* = 0.189, *t* = 20.91). This result implies that executive overconfidence can indirectly improve environmental technology innovation by promoting digital transformation. In order to ensure the reliability of the conclusion, we further adopted the Sobel test and Bootstrapping. The Sobel Z statistic is 13.91 and is significant at the level of 1%, which is further verified by Bootstrapping’s results. These results prove that digital transformation plays a significant partially mediating role in the relationship between executive overconfidence and environmental technology innovation, thus, supporting our hypothesis H3a.

Columns (6) and (7) of Table 4 show the regression results of the influence of digital transformation on environmental management innovation (*EMI*) and the mediating role of digital transformation. The coefficient of *DIG* shown in Column (6) of Table 4 is 0.010 (*t* = 3.41), which is statistically positive and significant at the 1% level. The results in Column (7) of Table 4 show that the coefficients of *DIG* are still significantly positive at the 1% level (*DIG*: *β* = 0.010, *t* = 3.29); however, the coefficient of *OC* is not significant. This result demonstrates that executive overconfidence can indirectly improve environmental management innovation by promoting digital transformation, lending support to our predictions in H3b.

### 4.3. Robustness Tests

#### 4.3.1. Propensity Score Matching

To alleviate the endogeneity concern, we adopted the Propensity Score Matching (PSM) method to eliminate the influence of sample selection bias [68]. First, we used companies with overconfident executives as the treatment group (*OC* = 1) and those without as the control group (*OC* = 0). Then, one-to-one matching of the nearest neighbors within the caliper radius (0.01) was performed according to the matching variables: *LEV*, *ROA*, *SIZE*, *Growth*, *CASH*, *Tobin’s Q*, *SOE*, *AGE*, *DUAL*, *IND*, and *BOARD*. The matching estimates for the average treatment effect (ATT) of *OC* shown in Panel A of Table A2 in Appendix A are all significantly positive, indicating that there were significant differences between the outcomes of the treatment group and of the control group. Next, we performed balance tests, and we present the results in Appendix A. As shown in Panel A of Table A3, the standardized biases of the most control variables were reduced after matching, and the t-test results did not reject the original hypothesis that there was no systematic difference between the two groups. After the balance test, the paired samples were taken into model (1) and model (2) for regression analysis, and the results are shown in Columns (1) to (3) of Table 5. The coefficients of *OC* are still significant at the 1% level when the dependent variable is *ETI* or *DIG*, which is consistent with the previous conclusion.

Second, we classified the samples with *DIG* values higher than the median into the treatment group (*DIG_D* = 1), and the other samples into the control group (*DIG_D* = 0). Then, we rematched the samples according to the above matching variables and matching methods, and finally obtained 16,543 matched samples. There were also significant differences in the ATT between the outcomes of the treatment group and of the control group shown in Panel B of Table A2. As shown in Panel B of Table A3, the sample was balanced after matching. The matched samples were then used to perform regression analysis based on model (3). Columns (4) and (5) of Table 5 show the final results. For the dependent variables *ETI* and *EMI*, the *DIG* coefficients are 0.149 and 0.013, respectively, both of which are significant at the 1% level, showing no significant difference with the original regression results. Overall, the results remain stable under the PSM test.

#### 4.3.2. Heckman Two-Stage Model

In order to address the problem of sample selection bias, we further adopted the Heckman two-stage model for the robustness test. In the first stage, we set up dummy variables *DIG_D*, *ETI_D*, and *EMI_D*, respectively, to measure whether an enterprise implements digital transformation, environmental technology innovation, and environmental management innovation, and then performed logit regression, in which *LEV*, *ROA*, *SIZE*, *Growth*, *CASH*, *Tobin’s Q*, *SOE*, *AGE*, *DUAL*, *IND*, and *BOARD* were controlled. Based on the results, we calculated the inverse Mills ratio (*IMR*) of the three models respectively: *IMR_DIG*, *IMR_ETI*, and *IMR_EMI*. 

In the second stage, we placed *IMR* into the corresponding regression models as a control variable, and the regression results are shown in Table 6. The results show that the *IMR* coefficients calculated in the first stage are significantly negative, indicating that selection bias did exist in the original regression analysis. However, the coefficients of the independent variables in Column (1), and Columns (3) to (5) of Table 6 are still significant at the 1% level after controlling for the sample selection bias, proving that the previous regression results are still robust.

#### 4.3.3. Sensitivity Tests

We further provided additional sensitivity tests. Table 7 displays an overview of the robustness tests undertaken and summarizes the pertinent results. Our main conclusions that executive overconfidence can enhance environmental innovation by promoting digital transformation continue to hold for all modifications.

First, we changed the measurement of key variables. We referred to Schrand and Zechman [61] and designated executives as overconfident if the firm’s residual from a regression of total asset growth on sales growth was less than the industry median residual. When the excess investment was greater than the industry median, *OC* was assigned a value of 1; otherwise, it was 0. As for digital transformation, we remeasured the degree of digital transformation by the total number of keyword frequencies. Columns (1) to (5) of Table 7 report the regression results after changing the measurement methods of executive overconfidence and digital transformation, which are consistent with the main conclusions.

Second, we narrowed the annual range to 2012–2019 to obtain sub-sample I. Since the global financial crisis broke out in 2008, the operating income and environmental innovation activities of listed companies experienced abnormal fluctuations, which continued until 2011. In order to avoid the perjury caused by the outlier effects listed above, we performed the empirical test again with the modified sample and present the results in Columns (6) to (10) of Table 7. Despite the large reduction in sample size, the results are qualitatively consistent with the previous conclusions.

Third, we selected the manufacturing industry as the sub-sample II to test our hypotheses again. This is because manufacturing accounts for about 63 percent of our full sample, which is the core of the real economy. In addition, China’s manufacturing industry is facing the pressure of transformation and upgrading, and its digital transformation and environmental innovation process may be different from other industries. The results are shown in Columns (11) to (15) of Table 7, which are consistent with the previous conclusions.

Fourth, we addressed the time-lag effects of executive overconfidence and digital transformation. We lagged the *OC* and *DIG* by 1 year and 2 years, respectively, and then conducted regression analysis. The results are shown in Columns (16) to (25) of Table 7. According to the results, the influence of executive overconfidence on environmental technology innovation and the mediating role of digital transformation is more significant after a lag of one and two years. In addition, the impact of digital transformation on environmental management innovation is also more significant after a lag of one and two years.

Fifth, we included firm fixed effect in the model to control for the omission of variables that vary with firms but not over time. Since the above test shows that the lagged digital transformation has a more significant impact on environmental management innovation, we made a lag treatment for digital transformation and then conducted fixed-effects regression. Columns (26) to (30) of Table 7 report the regression results after the change in the regression model. The results demonstrate that the original conclusions are still robust after controlling for firm fixed effects.

### 4.4. Further Analysis

Thus far, our results suggest that executive overconfidence can positively affect environmental innovation by influencing digital transformation. In order to further explore the impacts of contingent factors, we introduced moderating effect tests on the relationships between executive overconfidence and digital transformation and between digital transformation and environmental innovation. Table 8 presents the results of the moderating effects tests.

First, we explored the external factors that can regulate the relationship between executive overconfidence and digital transformation from the perspectives of industry and the macro environment. As shown in Column (1) of Table 8, the coefficient for the interaction term *OC* × *HHI* is significantly negative at the 1% level. Since the lower the *HHI*, the more intense the competition, this result means that the pressure of industry competition strengthens the promotion effect of executive overconfidence on digital transformation. Therefore, H4 is valid.

Column (2) of Table 8 shows the regression results after the introduction of the interaction term *OC* × *EPU*. The coefficient of *OC* × *EPU* is positive and statistically significant at the 1% level, suggesting that, in the case of high economic uncertainty, overconfident executives play a more obvious role in promoting enterprise digital transformation. Hence, H5 is also supported. In general, overconfident executives faced with high industry competition and highly uncertain economic policies are better able to turn pressure into motivation and gain competitive advantages by promoting digital transformation.

Secondly, we explored the internal moderating factors that can influence the effect of digital transformation on environmental innovation. As presented in Columns (3) and (4) of Table 8, when the dependent variable is *ETI*, the coefficient of *DIG* × *SIZE* is significantly positive at the 1% level, while when the dependent variable is *EMI*, the coefficient of *DIG* × *SIZE* is not significant. This indicates that enterprise asset scale can only adjust the relationship between digital transformation and environmental technology innovation but has no significant effect on the relationship between digital transformation and environmental management innovation. Thus, H6a is tenable, whereas H6b is untenable.

In addition, Columns (5) and (6) of Table 8 reveal the moderating effect of internal control quality on the relationship between digital transformation and environmental innovation. The coefficient of *DIG* × *ICQ* in Column (5) of Table 8 is significantly positive at the 5% level, indicating that the positive effect of digital transformation on environmental technology innovation is more prominent in the high-quality internal control environment. In addition, the coefficient of *DIG* × *ICQ* in Column (6) of Table 8 is significantly negative on the contrary, implying that the positive effect of digital transformation on environmental management innovation is more prominent in the weak internal control environment. Thus, H7a and H7b are both supported.

## 5. Discussion

The purpose of this study is to present an integrated model that examines the relationship among the three basic elements of people (executive overconfidence), technology (digital transformation), and innovation (environmental innovation). Taking inspiration from previous research, we explored the impact of executive overconfidence on environmental innovation by analyzing the roles of digital transformation. Furthermore, we examined the moderating effects of industrial competition, economic policy uncertainty, asset size, and internal control quality.

Our main results show that executive overconfidence had a significant positive effect on environmental technology innovation; however, it had no significant effect on environmental management innovation. This result further supports the research of He et al. [8] and Zhou et al. [35], suggesting that executives’ experience (e.g., academic experience) and personal traits (e.g., age and gender) can significantly influence their cognitive and psychological characteristics, thus, affecting environmental innovation. However, according to Ren et al. [1], the uncertainty and risk of environmental management innovation are relatively lower compared with those of environmental technology innovation. Perhaps for this reason, overconfident executives with higher risk preferences are more inclined to engage in environmental technology innovation rather than management innovation. In addition, overconfident executives tend to be autocratic and ignore the importance of internal control, which has some negative effects on enterprise management, such as negatively affecting carbon information disclosure [13]. These negative effects may offset the positive effects of overconfidence on environmental management innovation, thus, explaining our results.

Regarding the mediating role of digital transformation, our findings show that overconfident executives can significantly enhance digital transformation, and digital transformation can further promote environmental technology innovation and environmental management innovation. This finding is in line with Li and Shen [10]’s suggestions that digital transformation promoted by overconfident executives is an effective path to enhancing environmental innovation. In addition, the impact of digital transformation on environmental management innovation often has a time-lagged effect. This indicates that the impact of digital transformation on environmental management innovation may be hindered by organizational inertia [54], which often takes some time to absorb.

To further validate our assumptions, we selected industry competition (*HHI*) and economic policy uncertainty (*EPU*) as the moderating variables of the relationship between executive overconfidence and digital transformation. Specifically, in the context of higher industrial competition intensity and higher uncertainty of economic policies, executive overconfidence played a more significant role in promoting digital transformation, consistent with H4 and H5. This can be explained that in the face of external competitive pressure and uncertainty, overconfident executives are more capable of coping with challenges and are more committed to implementing digital transformation to help enterprises break through the dilemma.

Then, we choose asset size (*SIZE*) and internal control quality (*ICQ*) as the moderating variables of the relationship between digital transformation and environmental innovation. According to the empirical results, enterprise asset size can only adjust the relationship between digital transformation and environmental technology innovation but has no significant moderating effect on the relationship between digital transformation and environmental management innovation. The results confirm H6a and negate H6b. This can be explained by the fact that environmental management innovation usually requires fewer resources and less time than environmental technology innovation [1], which means it is less subject to competition for organizational resources with digital transformation [2]. 

As for the moderating effect of internal control, empirical results demonstrate that the positive effect of digital transformation on environmental technology innovation is more significant in the strong internal control environment, while the positive effect of digital transformation on environmental management innovation is more significant in the weak internal control environment, and these findings are consistent with H7a and H7b. Our research further confirms that digital transformation and corporate governance are substitutes for each other [10]. However, this substitution effect is only effective for enterprises’ environmental management innovation. As for environmental technology innovation, digital transformation not only promotes technological innovation by improving corporate governance but also by enhancing market agility and resource integration efficiency, and thus their relationship can be more significant in the case of high-level corporate governance [9].

### 5.1. Theoretical Implications

Our study makes several contributions to theoretical research on upper echelon theory, environmental innovation, and digital transformation. First, we reveal the influence channels of executive psychological characteristics on environmental innovation, which further enriches upper echelon theory. Most previous studies suggested that executive overconfidence has a negative effect on enterprise performance [13,61]. However, our study breaks this view and affirms the positive effect of such psychological traits on enterprise digital transformation and environmental innovation. Overconfident executives tend to have an optimistic attitude and strong resilience. They can take the initiative to meet challenges and help enterprises rise from adversity through transformation and innovation. This further expands the application of upper echelon theory in the new era of major changes and adjustments.

Second, our detailed analysis complements the research on the influencing factors of environmental innovation. Environmental innovation provides a fresh impetus for the sustainable development of enterprises and is of great significance to the improvement of economic performance and environmental performance. Our study elaborates on the impact of executive overconfidence and digital transformation on environmental technology innovation and environmental management innovation. In addition, we also introduce the moderating role of external factors, such as industry competition and economic policy uncertainty and internal factors, such as enterprise asset size and internal control quality. These findings broaden the scope of theoretical research on environmental innovation.

Third, our research enriches the research on digital transformation. In the digital era, the implementation of digital transformation is an irresistible trend for enterprises, and thus it has become a current research hotspot. While previous research has pointed to the important role of human resources in digital transformation, it has yet to reveal what kind of talent drives high-quality transformation. Our study extends previous literature and demonstrates the positive impact of overconfident executives on digital transformation. At the same time, we also verified the positive effects of digital transformation on environmental innovation, which provides a theoretical basis for enterprises to improve their environmental performance through digital transformation.

### 5.2. Practical Implications

Our empirical findings provide several practical implications. First, our conclusions provide references for the selection and employment of corporate executives. Enterprises should pay more attention to the psychological quality of executives in the process of establishing a management team. Especially in the macro environment full of uncertainty and competition, enterprises should change the stereotyped perception of executive overconfidence, and acknowledge the positive role of overconfidence in resisting risks, coping with changes, and achieving growth. Enterprises should select or replace overconfident executives reasonably according to their development stage, performance, and strategic needs, and formulate corresponding restraint and incentive systems.

Second, in the face of the digital wave, enterprises should strengthen their courage to change and increase their investments in digital transformation and environmental innovation. Enterprises should attach great importance to the deep integration of digital orientation and environmental orientation to thereby enhance the sustainability of enterprises by promoting environmental innovation with digital technology. Enterprises, especially small and medium-sized enterprises with resource constraints, should pay more attention to reconfiguring and adjusting organizational resources according to their resource endowment and development needs to adapt to the strategic path.

Third, since the positive effect of digital transformation on environmental technology innovation can be enhanced by internal control quality, enterprises can optimize the management process and strengthen internal supervision through digital transformation, to thus improve the quality of their internal control. In addition, enterprises can organically combine digital goals and environmental goals through the top-level design of internal management processes and thereby enhance their environmental management innovation.

## 6. Conclusions and Limitations

### 6.1. Conclusions

This study investigated the relationship among executive overconfidence, digital transformation, and environmental innovation. We conducted an empirical test using the panel data of Chinese publicly listed enterprises during the period 2007–2019. Our findings suggested that executive overconfidence has a positive effect on environmental innovation, especially on environmental technology innovation. In addition, digital transformation is an effective way for executives to improve their environmental innovation, including environmental technology innovation and environmental management innovation. 

The results of the moderating effect test showed that industry competition and economic policy uncertainty can enhance the positive effect of executive overconfidence on digital transformation. Moreover, the firm asset size can enhance the impact of digital transformation on environmental technology innovation. Internal control positively moderates the impact of digital transformation on environmental technology innovation and negatively moderates the impact on environmental management innovation.

### 6.2. Limitations and Future Research

This study also has some inherent limitations that need to be overcome in the future. First, our research focused mainly on Chinese listed companies; therefore, there are certain limitations in the generalizability of our conclusions. Given the different institutional contexts and economic environment between countries, our findings may be more applicable to similar developing countries rather than all countries. Future studies could re-examine the applicability of our research model in various contexts. 

Second, the measurement of digital transformation still needs to be refined. At present, we measured the degree of digital transformation of enterprises according to their use of typical digital technologies and facilities; however, this cannot fully represent the degree of integration of digitalization and business. We still need to conduct in-depth research to establish comprehensive and systematic digital quantitative indicators. 

Third, our quantitative research method is relatively weak in interpretation, and we plan to adopt qualitative methods in future research, such as case analysis and qualitative comparative analysis (QCA). Future research can adopt qualitative research techniques to explore the impact of different psychological characteristics of executives on digital transformation and environmental innovation in different situations. In addition, we will collect more comprehensive data through questionnaire surveys and case studies to further characterize the influence mechanism between digital transformation and environmental innovation.

## Figures and Tables

**Table 1:** Items and keywords of digital transformation.

Items	Keywords of Digital Transformation
Artificial Intelligence	“Business intelligence”, “Artificial intelligence”, “Machine learning”, “Business intelligence”, “Intelligent robot”, “Deep learning”, “Face recognition”, “Image understanding”, “Intelligent data analysis”, “Intelligent transportation”…
Blockchain	“Blockchain”, “Digital currency”, “Intelligent contract”, “League chain”, “Distributed computing”, “Consensus mechanism”, “Bitcoin”, “Digital currency”…
Data Management	“Big data analysis”, “Data mining”, “Text mining”, “Heterogeneous data”, “Data visualization”, “Distributed database”, “Virtual reality”, “Augmented reality”…
Multichannel	“O2O”, “C2C”, “C2B”, “B2B”, “B2C”, “Online retail”, “Online”, “E-business”, “E-commerce”, “Digital marketing” …
Digital Infrastructure	“5G”, “The Internet of things”, “The Internet”, “Industrial Internet”, “Cloud computing” …

**Table 2:** Variable definitions.

Variables	Definition
Independent Variable	
*OC*	Indicator variable that is equal to 1 if CEO is overconfident in a given year, and 0 otherwise.
Dependent Variables	
*ETI*	Natural logarithm of 1 plus the aggregate number of green patents filed in the application.
*EMI*	Indicator variable that is equal to 1 if the firm has ISO14001 certification in a given year, and 0 otherwise.
Mediating variable	
*DIG*	An ordered classification variable, equal to 0–5 according to the relevant statements in the company’s annual report.
Control Variables	
*LEV*	The ratio of total liabilities to total assets at the end of the fiscal year.
*ROA*	The ratio of net income to total assets at the end of the fiscal year.
*Growth*	The ratio of change in the current year’s sales revenue relative to last year’s sales revenue.
*Cash*	The ratio of cash and equivalents to total assets at the end of the fiscal year.
*Tobin’s Q*	The ratio of market value to book value at the end of the fiscal year.
*SOE*	Indicator variable that is equal to 1 if a firm is controlled by the state, and 0 otherwise.
*AGE*	The natural log of a firm’s age.
*DUAL*	Indicator variable that is equal to 1 if the CEO and the chairman of the board are the same people, and 0 otherwise.
*IND*	Percentage of independent (outside) directors on the board.
*BOARD*	The natural log of the number of members on the board of directors.
*GDP*	The percentage change from the preceding period in per capita real GDP.
*Market*	The natural log of the level of marketization in the listed company’s province.

**Table 3:** Summary and correlation statistics.

Panel A: Distributional Properties								
Variables	N	Mean	Sd	P1	P25	P50	P75	P99
A: *OC*	22,989	0.267	0.442	0	0	0	1	1
B: *DIG*	22,989	0.785	1.085	0	0	0	1	4
C: *ETI*	22,989	0.807	1.165	0	0	0	1.386	4.522
D: *EMI*	22,989	0.198	0.398	0	0	0	0	1
E: *LEV*	22,989	0.457	0.210	0.0594	0.293	0.454	0.613	0.950
F: *ROA*	22,989	0.0335	0.0650	−0.292	0.0121	0.0332	0.0619	0.197
G: *Growth*	22,989	6.966	893.3	−0.568	−0.0221	0.106	0.267	3.038
H: *CASH*	22,989	0.549	0.208	0.0855	0.405	0.561	0.705	0.956
I: *Tobin’s Q*	22,989	2.180	4.245	0.876	1.227	1.609	2.346	8.752
G: *SOE*	22,989	0.449	0.497	0	0	0	1	1
K: *AGE*	22,989	2.160	0.736	0.693	1.609	2.303	2.773	3.219
L: *DUAL*	22,989	0.226	0.418	0	0	0	0	1
M: *IND*	22,989	0.373	0.0556	0.333	0.333	0.333	0.429	0.571
N: *BOARD*	22,989	2.148	0.203	1.609	1.946	2.197	2.197	2.708
O: *GDP*	22,989	7.625	1.288	6.109	6.752	7.042	7.860	10.64
P: *Market*	22,989	2.182	0.242	1.327	2.113	2.224	2.345	2.442
**Panel B: Correlations Part I**								
**Variables**	**A**	**B**	**C**	**D**	**E**	**F**	**G**	**H**
A: *OC*	1							
B: *DIG*	0.217 ***	1						
C: *ETI*	0.081 ***	0.212 ***	1					
D: *EMI*	0.029 ***	0.038 ***	0.122 ***	1				
E: *LEV*	−0.156 ***	−0.132 ***	0.114 ***	−0.051 ***	1			
F: *ROA*	0.034 ***	0.023 ***	0.015 **	0.042 ***	−0.353 ***	1		
G: *Growth*	−0.00300	−0.00500	−0.00500	−0.00400	0.00700	0.00100	1	
H: *CASH*	0.123 ***	0.140 ***	0.048 ***	0.026 ***	−0.021 ***	0.105 ***	−0.00700	1
I: *Tobin’s Q*	0.030 ***	0.0100	−0.060 ***	−0.030 ***	−0.057 ***	0.026 ***	−0.00100	0.044 ***
G: *SOE*	−0.250 ***	−0.205 ***	0.00600	−0.042 ***	0.266 ***	−0.062 ***	0.00600	−0.165 ***
K: *AGE*	−0.277 ***	−0.086 ***	0.014 **	−0.058 ***	0.318 ***	−0.132 ***	0.00600	−0.142 ***
L: *DUAL*	0.439 ***	0.119 ***	−0.00100	0.00100	−0.122 ***	0.019 ***	−0.00200	0.084 ***
M: *IND*	0.060 ***	0.071 ***	0.051 ***	−0.012 *	−0.012 *	−0.029 ***	−0.00500	0.041 ***
N: *BOARD*	−0.128 ***	−0.104 ***	0.036 ***	0.023 ***	0.150 ***	0.032 ***	0.00200	−0.140 ***
O: *GDP*	−0.080 ***	−0.361 ***	−0.232 ***	−0.114 ***	0.148 ***	0.039 ***	0.00200	−0.037 ***
P: *Market*	0.128 ***	0.214 ***	0.130 ***	0.049 ***	−0.128 ***	0.049 ***	−0.012 *	0.116 ***
**Panel C: Correlations Part II**								
**Variables**	**I**	**G**	**K**	**L**	**M**	**N**	**O**	**P**
I: *Tobin’s Q*	1							
G: *SOE*	−0.064 ***	1						
K: *AGE*	0.012 *	0.414 ***	1					
L: *DUAL*	0.032 ***	−0.286 ***	−0.203 ***	1				
M: *IND*	0.022 ***	−0.067 ***	−0.029 ***	0.098 ***	1			
N: *BOARD*	−0.072 ***	0.281 ***	0.111 ***	−0.176 ***	−0.485 ***	1		
O: *GDP*	0.019 ***	0.213 ***	−0.021 ***	−0.108 ***	−0.087 ***	0.149 ***	1	
P: *Market*	−0.00900	−0.191 ***	−0.105 ***	0.119 ***	0.019 ***	−0.115 ***	−0.286 ***	1

Note: ***, **, and * represent significance levels at 1 percent, 5 percent, and 10 percent, respectively.

**Table 4:** The main empirical results.

Variables	(1)	(2)	(3)	(4)	(5)	(6)	(7)
	H1a: *ETI*	H1b: *EMI*	H2: *DIG*	H3a: *ETI*	H3a: *ETI*	H3b: *EMI*	H3b: *EMI*
*OC*	0.211 ***	0.009	0.258 ***		0.162 ***		0.007
	(10.97)	(1.32)	(15.44)		(8.56)		(0.94)
*DIG*				0.196 ***	0.189 ***	0.010 ***	0.010 ***
				(21.84)	(20.91)	(3.41)	(3.29)
*LEV*	1.176 ***	0.011	0.124 ***	1.148 ***	1.153 ***	0.010	0.010
	(28.72)	(0.81)	(3.66)	(28.61)	(28.79)	(0.71)	(0.72)
*ROA*	1.532 ***	0.249 ***	0.234 **	1.477 ***	1.488 ***	0.246 ***	0.246 ***
	(13.57)	(6.23)	(2.18)	(13.37)	(13.49)	(6.16)	(6.17)
*Growth*	−0.000 ***	−0.000 ***	0.000 ***	−0.000 ***	−0.000 ***	−0.000 ***	−0.000 ***
	(−10.86)	(−4.81)	(3.48)	(−10.99)	(−11.24)	(−4.91)	(−4.91)
*CASH*	0.309 ***	0.004	0.469 ***	0.235 ***	0.221 ***	−0.000	−0.001
	(7.71)	(0.27)	(13.91)	(5.92)	(5.58)	(−0.03)	(−0.07)
*Tobin’s Q*	−0.012 **	−0.002 **	−0.007 **	−0.011 **	−0.011 **	−0.002 **	−0.002 **
	(−2.39)	(−2.33)	(−2.26)	(−2.31)	(−2.33)	(−2.30)	(−2.31)
*SOE*	0.129 ***	0.016 ***	−0.162 ***	0.156 ***	0.160 ***	0.018 ***	0.018 ***
	(7.36)	(2.66)	(−11.51)	(9.04)	(9.26)	(2.90)	(2.92)
*AGE*	0.026 **	−0.016 ***	0.009	0.011	0.024 **	−0.017 ***	−0.016 ***
	(2.23)	(−3.77)	(0.89)	(0.94)	(2.12)	(−3.96)	(−3.79)
*DUAL*	−0.133 ***	−0.028 ***	−0.025	−0.065 ***	−0.129 ***	−0.025 ***	−0.028 ***
	(−6.94)	(−3.96)	(−1.48)	(−3.82)	(−6.83)	(−3.84)	(−3.92)
*IND*	1.445 ***	0.029	0.695 ***	1.316 ***	1.314 ***	0.022	0.022
	(8.55)	(0.56)	(5.94)	(7.83)	(7.84)	(0.43)	(0.43)
*BOARD*	0.473 ***	0.104 ***	0.122 ***	0.446 ***	0.449 ***	0.102 ***	0.102 ***
	(10.37)	(7.05)	(3.58)	(9.95)	(10.06)	(6.95)	(6.97)
*GDP*	−0.109 ***	−0.022 ***	−0.126 ***	−0.086 ***	−0.085 ***	−0.020 ***	−0.020 ***
	(−25.79)	(−14.88)	(−31.80)	(−20.52)	(−20.44)	(−13.65)	(−13.64)
*Market*	0.497 ***	0.049 ***	0.261 ***	0.459 ***	0.448 ***	0.047 ***	0.046 ***
	(15.31)	(3.97)	(10.95)	(14.63)	(14.36)	(3.81)	(3.77)
Constant	−1.922 ***	0.052	0.541 ***	−2.006 ***	−2.024 ***	0.047	0.238 ***
	(−11.28)	(0.90)	(4.28)	(−11.97)	(−12.11)	(0.82)	(6.54)
Year FE	YES	YES	YES	YES	YES	YES	YES
Industry FE	YES	YES	YES	YES	YES	YES	YES
Adjusted-R2	0.197	0.0626	0.351	0.214	0.217	0.0631	0.0631
Observations	22,989	22,989	22,989	22,989	22,989	22,989	22,989
Sobel-test				0.04870 ***		0.00261 ***	
				(13.91)		(3.402)	
Bootstrap r(ind_eff)				0.04870 ***		0.00261 ***	
[95% Conf. Interval]				[0.04071 ⋯ 0.05670]		[0.00092 ⋯ 0.00430]	
Bootstrap r(dir_eff)				0.16198 ***		0.00658	
[95% Conf. Interval]				[0.12583 ⋯ 0.19813]		[−0.00683 ⋯ 0.02000]	

Note: *** and ** represent significance levels at 1 percent and 5 percent, respectively; Robust t-statistics, clustered at the firm-level, are presented in parentheses.

**Table 5:** Endogenous test of propensity score matching.

Variables	(1)	(2)	(3)	(4)	(5)
	*ETI*	*EMI*	*DIG*	*ETI*	*EMI*
*OC*	0.208 ***	−0.003	0.236 ***		
	(9.11)	(−0.39)	(11.77)		
*DIG*				0.149 ***	0.013 ***
				(15.08)	(3.72)
*LEV*	1.353 ***	0.047 **	0.121 **	1.332 ***	0.019
	(19.47)	(2.03)	(2.08)	(26.92)	(1.08)
*ROA*	1.421 ***	0.334 ***	−0.272	1.490 ***	0.254 ***
	(7.63)	(5.29)	(−1.56)	(10.47)	(5.10)
*Growth*	−0.000 ***	−0.000	−0.000 ***	−0.000	−0.000 *
	(−2.83)	(−0.78)	(−4.03)	(−0.41)	(−1.86)
*CASH*	0.418 ***	−0.006	0.654 ***	0.156 ***	0.006
	(6.05)	(−0.27)	(10.96)	(3.17)	(0.37)
*Tobin’s Q*	−0.006 **	−0.001 **	−0.004	−0.010 *	−0.002 *
	(−2.02)	(−2.02)	(−1.41)	(−1.93)	(−1.86)
*SOE*	0.072 **	0.023**	−0.152 ***	0.229 ***	0.012
	(2.39)	(2.18)	(−6.22)	(10.96)	(1.60)
*AGE*	0.060 ***	−0.004	0.024	0.020	−0.014 ***
	(3.37)	(−0.67)	(1.53)	(1.51)	(−2.78)
*DUAL*	−0.120 ***	−0.043 ***	−0.036 *	−0.077 ***	−0.021 ***
	(−4.99)	(−4.88)	(−1.71)	(−3.94)	(−2.82)
*IND*	1.241 ***	0.105	0.954 ***	1.413 ***	−0.006
	(4.36)	(1.26)	(4.60)	(6.86)	(−0.10)
*BOARD*	0.486 ***	0.149 ***	0.151 **	0.401 ***	0.094 ***
	(6.15)	(5.94)	(2.37)	(7.17)	(5.33)
*GDP*	−0.228 ***	−0.048 ***	−0.319 ***	−0.085 ***	−0.019 ***
	(−15.26)	(−8.07)	(−26.99)	(−17.96)	(−11.00)
*Market*	0.579 ***	0.078 ***	0.420 ***	0.486 ***	0.045 **
	(10.74)	(3.55)	(7.94)	(11.94)	(2.56)
Constant	−1.568 ***	0.008	1.089 ***	−2.121 ***	0.075
	(−4.80)	(0.07)	(4.04)	(−10.13)	(1.03)
Year FE	YES	YES	YES	YES	YES
Industry FE	YES	YES	YES	YES	YES
Adjusted-R^2^	0.182	0.0541	0.365	0.217	0.0608
Observations	9063	9063	9063	16,543	16,543

Note: ***, **, and * represent significance levels at 1 percent, 5 percent, and 10 percent, respectively; Robust t-statistics, clustered at the firm-level, are presented in parentheses.

**Table 6:** Endogenous test of the Heckman two-stage model.

Variables	(1)	(2)	(3)	(4)	(5)
	*ETI*	*EMI*	*DIG*	*ETI*	*EMI*
*OC*	0.191 ***	0.007	0.248 ***		
	(10.26)	(0.96)	(14.91)		
*DIG*				0.158 ***	0.009 ***
				(18.33)	(2.90)
*IMR_DIG*			−0.525 ***		
			(−16.49)		
*IMR_ETI*	−1.028 ***			−0.971 ***	
	(−40.53)			(−38.79)	
*IMR_EMI*		−0.291 ***			−0.289 ***
		(−14.19)			(−14.11)
*LEV*	0.467 ***	0.010	0.085 **	0.482 ***	0.009
	(11.88)	(0.72)	(2.53)	(12.40)	(0.64)
*ROA*	0.566 ***	0.007	0.048	0.573 ***	0.007
	(5.29)	(0.17)	(0.45)	(5.42)	(0.15)
*Growth*	0.000 ***	0.005 ***	0.000 ***	0.000 ***	0.004 ***
	(16.42)	(9.34)	(16.59)	(16.09)	(9.22)
*CASH*	0.051	−0.015	0.189 ***	0.007	−0.018
	(1.31)	(−1.03)	(5.01)	(0.19)	(−1.28)
*Tobin’s Q*	0.008 ***	0.010 ***	−0.001	0.008 ***	0.010 ***
	(5.95)	(6.88)	(−0.25)	(7.56)	(6.89)
*SOE*	0.015	−0.003	−0.095 ***	0.043 **	−0.001
	(0.90)	(−0.42)	(−6.49)	(2.55)	(−0.19)
*AGE*	0.059 ***	0.000	0.022 **	0.042 ***	−0.000
	(5.36)	(0.10)	(2.20)	(3.96)	(−0.02)
*DUAL*	−0.094 ***	−0.010	−0.052 ***	−0.033 **	−0.008
	(−5.07)	(−1.33)	(−3.08)	(−1.97)	(−1.16)
*IND*	0.861 ***	−0.024	0.268 **	0.790 ***	−0.030
	(5.35)	(−0.46)	(2.24)	(4.92)	(−0.57)
*BOARD*	0.114 ***	0.013	0.018	0.112 **	0.013
	(2.60)	(0.84)	(0.51)	(2.57)	(0.80)
*GDP*	0.004	−0.001	−0.110 ***	0.039 ***	0.001
	(0.39)	(−0.28)	(−7.48)	(3.66)	(0.25)
*Market*	−0.002	−0.004	0.089 ***	−0.003	−0.006
	(−0.07)	(−0.36)	(3.66)	(−0.12)	(−0.49)
Constant	0.834 ***	0.607 ***	1.621 ***	0.481 ***	0.590 ***
	(4.65)	(8.37)	(11.52)	(2.68)	(8.12)
Year FE	YES	YES	YES	YES	YES
Industry FE	YES	YES	YES	YES	YES
Adjusted-R^2^	0.256	0.0707	0.358	0.266	0.0710
Observations	22,988	22,908	22,988	22,988	22,908

Note: *** and ** represent significance levels at 1 percent and 5 percent, respectively; Robust t-statistics, clustered at the firm-level, are presented in parentheses.

**Table 7:** Sensitivity tests.

*OC*→*ETI*	(1) Alternative *OC* Measure	(6) Sub-Sample I Year: 2012–2019	(11) Sub-Sample II Manufacturing	(16) Lag Effect *OC*_*t*-1_	(21) Lag Effect *OC*_*t*-2_	(26) Fixed-Effects Estimator
*OC*	0.128 ***	0.221 ***	0.112 ***	0.210 ***	0.218 ***	0.083 ***
	(8.66)	(9.96)	(5.10)	(9.94)	(9.33)	(3.20)
Controls	YES	YES	YES	YES	YES	YES
Year FE	YES	YES	YES	YES	YES	YES
Industry FE	YES	YES	YES	YES	YES	YES
Adjusted-R^2^	0.195	0.188	0.261	0.201	0.205	0.1971
Observations	22,989	17,908	14,596	19,250	16,192	20,372
***OC*** **→*EMI***	**(2)** **Alternative *OC*** **Measure**	**(7)** **Sub-Sample** **I** **Year: 2012–2019**	**(12)** **Sub-Sample** **II** **Manufacturing**	**(17)** **Lag Effect** ***OC*_*t*-1_**	**(22)** **Lag Effect** ***OC*_*t*-2_**	**(27)** **Fixed-Effects Estimator**
*OC*	−0.007	0.009	−0.007	0.011	0.022 ***	−0.014
	(−1.30)	(1.16)	(−0.81)	(1.46)	(2.62)	(−1.21)
Controls	YES	YES	YES	YES	YES	YES
Year FE	YES	YES	YES	YES	YES	YES
Industry FE	YES	YES	YES	YES	YES	YES
Adjusted-R^2^	0.0626	0.0566	0.049	0.0633	0.0632	0.0217
Observations	22,989	17,908	14,596	19,250	16,192	20,372
***OC*** **→** ***DIG***	**(3)** **Alternative *OC* and *DIG* Measure**	**(8)** **Sub-Sample** **I** **Year: 2012–2019**	**(13)** **Sub-Sample** **II** **Manufacturing**	**(18)** **Lag Effect** ***OC*_*t*-1_**	**(23)** **Lag Effect** ***OC*_*t*-2_**	**(28)** **Fixed-Effects Estimator**
*OC*	1.719 ***	0.281 ***	0.164 ***	0.279 ***	0.290 ***	0.261 ***
	(7.27)	(14.01)	(9.15)	(15.20)	(14.35)	(9.87)
Controls	YES	YES	YES	YES	YES	YES
Year FE	YES	YES	YES	YES	YES	YES
Industry FE	YES	YES	YES	YES	YES	YES
Adjusted-R^2^	0.248	0.304	0.330	0.349	0.338	0.2517
Observations	22,989	17,908	14,596	19,250	16,192	20,372
***DIG*** **→*ETI***	**(4)** **Alternative *DIG*** **Measure**	**(9)** **Sub-Sample** **I** **Year: 2012–2019**	**(14)** **Sub-Sample** **II** **Manufacturing**	**(19)** **Lag Effect** ***DIG*_*t*-1_**	**(24)** **Lag Effect** ***DIG*_*t*-2_**	**(29)** **Fixed-Effects Estimator**
*DIG*	0.008 ***	0.195 ***	0.161 ***	0.215 ***	0.243 ***	0.047 ***
	(14.77)	(20.87)	(13.55)	(20.31)	(19.68)	(6.28)
Controls	YES	YES	YES	YES	YES	YES
Year FE	YES	YES	YES	YES	YES	YES
Industry FE	YES	YES	YES	YES	YES	YES
Adjusted-R^2^	0.205	0.206	0.272	0.219	0.225	0.199
Observations	22,989	17,908	14,596	19,250	16,192	20,372
***DIG*** **→*EMI***	**(5)** **Alternative *DIG*** **Measure**	**(10)** **Sub-Sample** **I** **Year: 2012–2019**	**(15)** **Sub-Sample** **II** **Manufacturing**	**(20)** **Lag Effect** ***DIG*_*t*-1_**	**(25)** **Lag Effect** ***DIG*_*t*-2_**	**(30)** **Fixed-Effects Estimator**
*DIG*	0.001 ***	0.009 ***	0.009 **	0.013 ***	0.016 ***	0.012 **
	(5.01)	(2.94)	(2.02)	(3.77)	(3.99)	(2.52)
Controls	YES	YES	YES	YES	YES	YES
Year FE	YES	YES	YES	YES	YES	YES
Industry FE	YES	YES	YES	YES	YES	YES
Adjusted-R^2^	0.0637	0.0570	0.0298	0.0639	0.0638	0.0139
Observations	22,989	17,908	14,596	19,250	16,192	13,848

Note: The arrow → represents the path, with the independent variable on the left and the dependent variable on the right; *** and ** represent significance levels at 1 percent and 5 percent, respectively; Robust t-statistics, clustered at the firm-level, are presented in parentheses.

**Table 8:** Moderating effect test.

Variables	(1)	(2)	(3)	(4)	(5)	(6)
	*DIG*	*DIG*	*ETI*	*EMI*	*ETI*	*EMI*
*OC*	0.327 ***	0.186 ***				
	(15.19)	(6.17)				
*DIG*			−1.263 ***	0.013	0.059	0.046 **
			(−9.02)	(0.31)	(0.91)	(2.49)
OC × *HHI*	−0.569 ***					
	(−5.32)					
OC × *EPU*		0.0004 ***				
		(2.53)				
DIG × *SIZE*			0.063 ***	−0.000		
			(9.91)	(−0.13)		
DIG × *ICQ*					0.020 **	−0.006 **
					(2.01)	(−1.99)
*HHI*	−0.029					
	(−0.54)					
*EPU*		0.007 ***				
		(15.84)				
*SIZE*			0.326 ***	0.019 ***		
			(37.56)	(6.65)		
*ICQ*					0.118 ***	0.019 ***
					(10.79)	(5.23)
Controls	YES	YES	YES	YES	YES	YES
Year FE	YES	YES	YES	YES	YES	YES
Industry FE	YES	YES	YES	YES	YES	YES
Adjusted-R^2^	0.352	0.351	0.313	0.0650	0.222	0.0639
Observations	22,989	22,989	22,989	22,989	22,989	22,989

Note: *** and ** represent significance levels at 1 percent and 5 percent, respectively; Robust t-statistics, clustered at the firm-level, are presented in parentheses.

## Data Availability

The data shown in this research are available on request from the corresponding author.

## Appendix A

**Table A1:** Industry distribution and sampling firms.

Industry	Industry Code	No. of Listed Companies	Obs.	Percent (%)
Farming, forestry, animal husbandry, and fishery	A1-A5	44	339	1.47
The mining industry	B6-12	68	597	2.60
Manufacturing	C13-43	1933	14,596	63.49
Electricity, heat, gas, and water production and supply	D44-46	104	869	3.78
The construction industry	E47-50	91	623	2.71
Wholesale and Retail	F51-52	179	1323	5.75
Transportation, warehousing, and postal services	G53-60	91	815	3.55
Accommodation and Catering	H61-62	11	89	0.39
Information transmission, software, and information technology services	I63-65	247	1274	5.54
The real estate industry	K70	151	1168	5.08
Leasing and business services	L71-72	55	254	1.10
Scientific research and technology services	M73-75	33	149	0.65
Water conservancy, environment, and public facilities management	N76-78	56	229	1.00
Residential services, repairs, and other services	O79-81	7	22	0.10
Education industry	P82	7	13	0.06
Health and social work	Q83-84	9	39	0.17
Culture, sports, and entertainment	R85-89	48	265	1.15
Other	S90	79	325	1.41
Total		3213	22,989	100.00

**Table A2:** The matching estimates for average treatment effect on the treated (ATT).

Panel A. Average Treatment Effect of *OC*				
Outcome	Treated	Controls	Difference	T-Value
*ETI*	1.0116	0.7308	0.2809	11.27 ***
*EMI*	0.2133	0.1941	0.0192	2.27 **
*DIG*	1.1328	0.7390	0.3938	16.33 ***
**Panel B. Average Treatment Effect of *DIG_D***				
Outcome	Treated	Controls	Difference	T-Value
*ETI*	1.0446	0.6891	0.3555	19.41 ***
*EMI*	0.2212	0.1946	0.0266	4.22 ***

Note: *** and ** represent significance levels at 1 percent and 5 percent, respectively.

**Table A3:** Balance test of propensity score matching.

Panel A. Balance Test of *OC*					
Variables	Match	Treated Group	Controlled Group	%Bias	*p*-Value
*LEV*	Unmatched	0.40227	0.4764	−36.1	0.000 ***
	Matched	0.42636	0.42406	1.1	0.590
*ROA*	Unmatched	0.03726	0.03222	7.7	0.000 ***
	Matched	0.03535	0.03563	−0.4	0.842
*SIZE*	Unmatched	21.905	22.325	−33.9	0.000 ***
	Matched	22.033	22.028	0.5	0.821
*Growth*	Unmatched	2.7946	8.4621	−0.8	0.670
	Matched	3.7	0.32241	0.5	0.303
*CASH*	Unmatched	0.59186	0.53363	29.3	0.000 ***
	Matched	0.58128	0.58321	−1.0	0.626
*Tobin’s Q*	Unmatched	2.3874	2.1034	6.4	0.000 ***
	Matched	2.3567	2.3713	−0.3	0.909
*SOE*	Unmatched	0.24198	0.52373	−60.6	0.000 ***
	Matched	0.31894	0.30529	2.9	0.160
*AGE*	Unmatched	1.8219	2.2834	−64.5	0.000 ***
	Matched	1.9756	1.9486	3.8	0.086 *
*DUAL*	Unmatched	0.53151	0.11534	99.3	0.000 ***
	Matched	0.3663	0.35969	1.6	0.513
*IND*	Unmatched	0.37897	0.37145	13.4	0.000 ***
	Matched	0.37439	0.37469	−0.5	0.800
*BOARD*	Unmatched	2.105	2.1636	−29.3	0.000 ***
	Matched	2.1273	2.1231	2.1	0.301
**Panel B. Balance Test of *DIG***					
**Variables**	**Match**	**Treated Group**	**Controlled Group**	**%Bias**	***p*-Value**
*LEV*	Unmatched	0.42574	0.48004	−26.1	0.000 ***
	Matched	0.44216	0.43985	1.1	0.466
*ROA*	Unmatched	0.036	0.03171	6.6	0.000 ***
	Matched	0.03631	0.03651	−0.3	0.831
*SIZE*	Unmatched	22.304	22.144	12.3	0.000 ***
	Matched	22.272	22.264	0.6	0.677
*Growth*	Unmatched	0.56982	11.785	−1.3	0.345
	Matched	0.60192	0.41226	0.0	0.455
*CASH*	Unmatched	0.58074	0.52523	27.2	0.000 ***
	Matched	0.56381	0.56727	−1.7	0.264
*Tobin’s Q*	Unmatched	2.1929	2.1687	0.6	0.668
	Matched	2.1938	2.1125	2.0	0.221
*SOE*	Unmatched	0.3385	0.53204	−39.8	0.000 ***
	Matched	0.3931	0.38622	1.4	0.364
*AGE*	Unmatched	2.0829	2.2192	−18.5	0.000 ***
	Matched	2.1234	2.1221	0.2	0.910
*DUAL*	Unmatched	0.2815	0.18444	23.1	0.000 ***
	Matched	0.23226	0.23974	−1.8	0.257
*IND*	Unmatched	0.37775	0.3702	13.6	0.000 ***
	Matched	0.37443	0.37494	−0.9	0.557
*BOARD*	Unmatched	2.126	2.1646	−19.1	0.000 ***
	Matched	2.1419	2.1394	1.2	0.424

Note: *** and * represent significance levels at 1 percent and 10 percent, respectively.
